# Supplementary material for: Tomographic near-eye displays
Source: Nat Commun. 2019 Jun 7;10:2497. doi: 10.1038/s41467-019-10451-2 (PMC6555831; doi:10.1038/s41467-019-10451-2)
Supplement: Supplementary file 1 — Supplementary Information [file 41467_2019_10451_MOESM1_ESM.pdf]

Supplementary Information

Tomographic Near-Eye Displays

Seungjae Lee et al.

Supplementary Note 1: Additional Analysis of Tomographic Displays

Derivation of Upper-bound Amplitude and Bit Depth

In order to analyze contrast and resolution limit of volumetric and tomographic displays, we define maximum and upper-bound amplitudes which are given by

$$
\begin{aligned}
MA(v, z_a) &= \max\left(\sum_{j \in (z_a - z_j) < \frac{1}{wv}} b_j L_j(v) H(v, z_a; z_j)\right) = L_m(v) \sum_{j \in (z_a - z_j) < \frac{1}{wv}} b_j H(v, z_a; z_j), \\
MA(v, z_a) &\leq UA(v, z_a) = G \sum_{j \in (z_a - z_j) < \frac{1}{wv}} b_j H(v, z_a; z_j),
\end{aligned}
\tag{1}
$$

where $MA(v, z_a)$ and $UA(v, z_a)$ are maximum and upper-bound amplitudes, respectively, which are referred to as the Fourier coefficients (spatial frequency: v) of retinal images when focal depth of human eye is z_a. The Fourier coefficients can be derived from the sum of transfer function $H(v, z_a; z_j)$ of focal plane images, $b_j L_j(v)$. The luminance of backlight, b_j, may vary according to focal planes when direct digital synthesis is applied. Considering the finite-aperture version of the depth of field, we select adequate focal plane images sufficiently near from the focal depth of human eye. The selected focal plane images support maximum displayable frequency higher than the interested spatial frequency, v. Note that all focal plane images have the identical maximum Fourier coefficient, $L_m(v)$, whose upper-bound is supposed to be a constant, G (Parseval's theorem). Finally, we derive the upper-bound amplitude that is proportional to the sum of transfer function.

As described in Supplementary Eq. 1, the upper-bound amplitude is a function related to the spatial frequency and the focal depth. The upper-bound amplitude can be used as evaluation criteria of the contrast and resolution limit. First, we suppose that the upper-bound amplitude of zero frequency is normalization constant. The normalization constant is the maximum luminance of display systems since the amplitude is maximized when the FSAB always turn on. Second, we calculate the ratio of the upper-bound amplitude to the normalization constant according to the spatial frequency. The ratio indicates how bright images of corresponding frequency can be observed through tomographic displays. If the ratio is close to zero, the brightness of the image could be too dim to be observed. Ideal display system would have constant upper-bound amplitude, 1, regardless of focal depth as well as spatial frequency.

The bit depth is also an important factor to evaluate display performance. For representation of full color 8 bit images, display system should have 24 bit depth. In tomographic displays, at least 24 bit depth is supported by the additional display panel. In fact, tomographic displays would have higher bit depth because we can modulate the signal intensity by modulating illumination time of the backlight. For instance, the signal intensity of zero spatial frequency could have approximately 79 times more depths that corresponds to the increase of 6.3 bit depth. Note that the bit depth increase becomes slow at the high spatial frequency because only a few adjacent focal planes are coupled. The number of coupled focal planes is derived based on the upper-bounds on the spatial frequency[1].

Using the same methodology, we can also analyze the bit depth of related prototypes[2,3] as shown in the manuscript. For the prototype of Rathinavel et al.[2], each focal plane has 1 bit depth while combination of adjacent focal planes gives exponential variation due to the binary illumination. The exponential increase in bit depth continues until at least 24 focal planes are coupled. For the prototype of Chang et al.[3], each focal plane has 8 bit depth. In this prototype, the increase tendency in bit depth is similar with that of tomographic displays.

Necessity of Black Frames

For multiple focal plane reconstruction, the focus-tunable lens is operated by periodic signal such as sinusoidal or triangle wave. We may suppose two methods for arrangement of focal plane images in the periodic cycles where every focal depth is scanned twice within a single period. The first one is to display half of focal plane images during the forward cycle, and display the other half of focal plane images during the backward cycle as shown in Supplementary Fig. 1. This method allows tomographic displays to increase the number of focal planes up to 280 focal planes, which is identical to that of Ratinavel et al.'s prototype[2]. The second one is to employ only half of periodic cycle for representation of volumetric scenes while the other half cycle remains as a black frame. Although the second method loses frame rate, the black frame insertion is important for this type of displays when moving scenes are played.

Tomographic displays synthesize multiple focal plane images via temporal multiplexing. If a single cycle for the multiplexing takes a time less than 1/60 seconds, human observers recognize the focal plane images as a volumetric scene. However, human observers could be more sensitive and recognize the artificial effects of tomographic displays when they are exposed to a moving scene without black frames. We provide a brief exposition of this phenomenon in Supplementary Fig. 1. Since human observer likely recognizes adjacent frame images simultaneously, even a slight movement of volumetric scenes becomes noticeable artifacts. The synthesized images have stripe patterns where focal plane images of adjacent frames are not fitted well. On the other hands, the stripe patterns are alleviated when black frames are inserted.

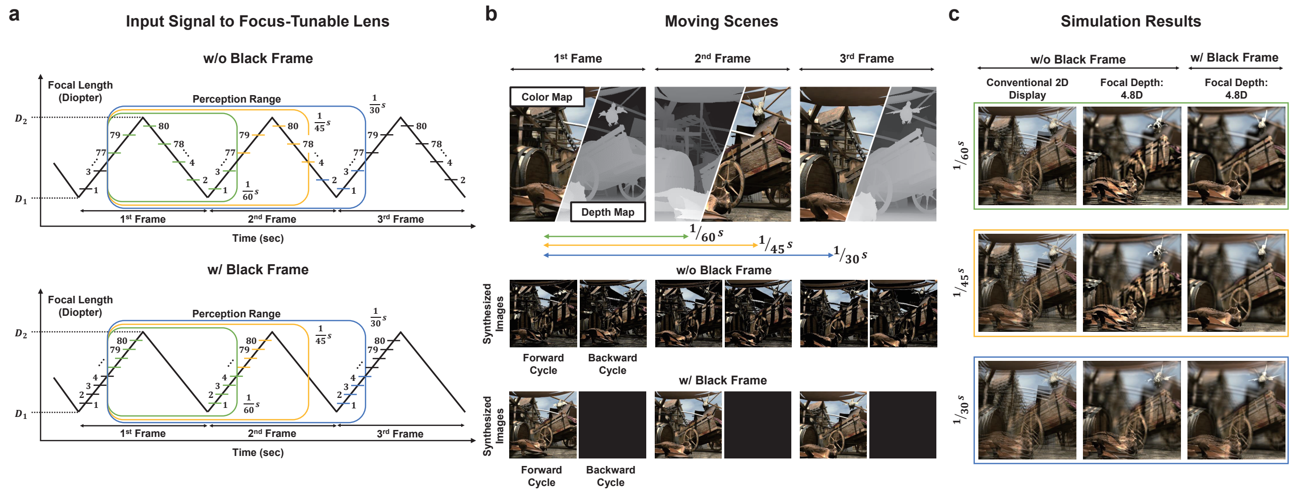

Supplementary Figure 1. Retinal image simulation of moving scenes. (a) We illustrate how focal plane images are synchronized with the focus-tunable lens when black frames are inserted. (b) We demonstrate how moving scenes are decomposed into periodic cycles of focus-tunable lens' signal. (c) Synthesized images are simulated in the various conditions to verify how the stripe patterns are recognized according to the exposure times. The results show that black frame insertion is efficient solution to alleviate the stripe pattern artifacts. With back frames, tomographic displays provide users with much more natural motion blur effect that is similar with conventional 2D displays.

Determination of the Number of Focal Planes

It has been an interesting topic to debate how many focal planes are necessary to reconstruct a volumetric object without noticeable artificial effects. According to perceptual studies[4], human visual system has limited depth of field (0.15D) so that users may not recognize the discrete structure of multi-plane system with layer spacing smaller than 0.15D. In tomographic displays, however, the layer spacing of 0.15D is not enough to conceal the discrete structure because of separation or overlap between adjacent focal plane images by the pupil movement. We note that more focal planes enable display system to have more tolerance for the pupil movement, which provides the intuition to determine the focal plane number of the benchtop prototype for tomographic near-eye displays.

Multi-focal system usually assumes that an observer's pupil is fixed at a specific point so that multi-plane images are properly synthesized at the retina. When the pupil is dislocated from the specific point, multi-plane images could be misaligned by relative disparity. This misalignment may cause the resolution loss or distortion of accommodation cues[5]. Accordingly, misalignment issue restricts the eye box of entire system as a fixed point. As limited eye box is not desired for near-eye displays, some advanced methods have been presented to expand the limited eye box with[6] or without a gaze-tracking system[5].

In tomographic displays, the misalignment of adjacent focal planes is alleviated when the number of focal planes is increased. As human visual system has the resolution limitation of 30 cycle per degree (cpd), tomographic displays with dense focal planes have some tolerance for pupil movement. When layer spacing of 0.058D is supported, users may not observe artifacts caused by misalignment of adjacent focal planes within 10mm. Our prototype of 0.07D layer spacing has tolerance for pupil movement up to 7.5mm, which is larger than the exit pupil determined by optical system. Detailed analysis and experimental results are demonstrated in following section.

In summary, dense focal planes improve the tolerance for the pupil movement. However, it could not resolve all challenging issues involved in representation of 3D scenes. If 3D scenes have large depth discontinuities, our prototype is also vulnerable to the pupil movement. For mitigation of the artifacts caused by depth discontinuities, we should adopt computational methods rather than optical solution. First, we may use approximated depth map with smooth variation, which was employed by Matsuda et al.[7]. We can also apply blending methods that optimize focal plane images to have more tolerance for depth discontinuities.

Misalignment by Pupil Movement

Supplementary Figure 2 illustrates how we analyze misalignment of focal planes in tomographic displays. When the pupil of observer is dislocated from the desired position, focal plane images are misaligned due to the disparity. Note that this misalignment is usually natural effect referred to as motion parallax. However, it causes artifacts including separation and overlap when multi-plane images reconstruct a volumetric object such as a slanted bar that extends along the several layers. In that case, the misalignment makes observers to feel that volumetric objects have discontinuous patterns.

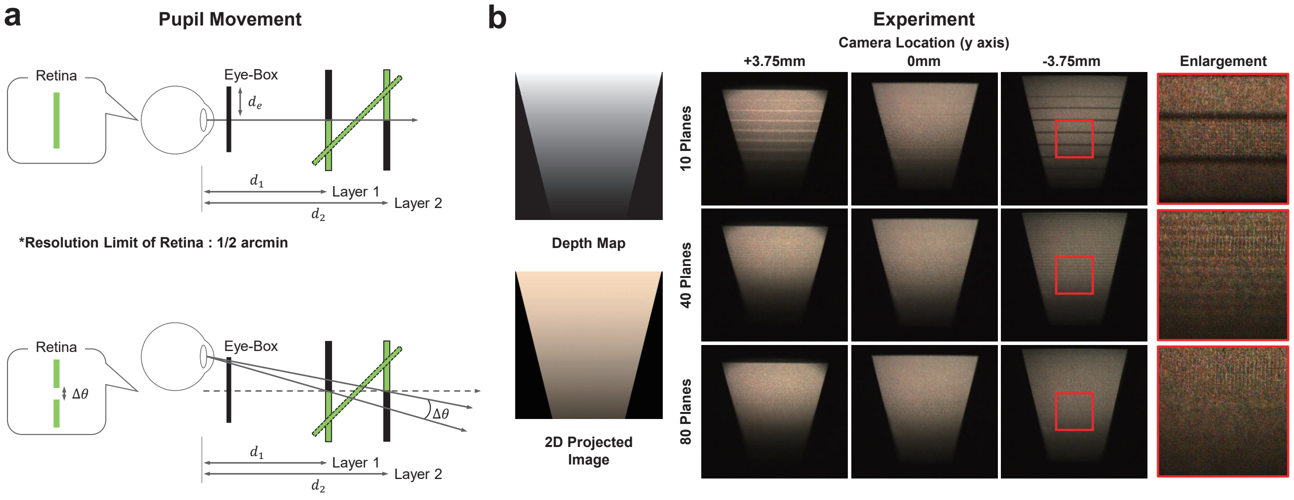

Supplementary Figure 2. Demonstration of pupil movement. (a) We illustrate how pupil movement affects alignment of adjacent focal planes. If separation of focal plane images is significantly small due to the dense focal planes, we can expect that human visual system may not notice the misalignment. (b) As shown in the experimental results, more layers may enlarge the tolerance for the pupil movement.

Since human visual system has a resolution limit about 30 cycles per degree (cpd), observer may not recognize the separation or overlap of adjacent plane images caused by pupil movement when the misalignment is small enough. In order to ensure that observer could not see this misalignment, layer spacing should be narrow enough. We can derive a relationship between the degree of misalignment and layer spacing as follows.

$$\Delta\theta = \tan^{-1}(d_e/d_1) - \tan^{-1}(d_e/d_2) \approx d_e\left(d_1^{-1} - d_2^{-1}\right). \tag{2}$$

When $\Delta\theta$ is less than 1/2 arcmin, human visual system could not perceive the misalignment. According to this equation, tomographic displays secure expanded tolerant region of 10mm when layer spacing is narrower than 0.058D. Our prototype with 0.07D spacing has tolerance for pupil movement of 7.5mm.

Supplementary Note 2: Details of Occlusion Blending for Tomographic Displays

For mitigation of artifact at occlusion boundary with the large depth discontinuities, we could apply occlusion blending method. As described in the manuscript, the optimization problem has more constraints compared to the related problem[1,8]. In this study, we designed a primitive solver for the optimization, which demonstrates the ability of tomographic displays to support occlusion effect.

Algorithm to Solve NP-hard Problem

We divided the optimization problem into two steps: 1) set $\mathbf{B}_k$ as constant and update $\mathbf{D}$. 2) set $\mathbf{D}$ as constant and update $\mathbf{B}_k$. We repeated two steps iteratively because both of $\mathbf{B}_k$ and $\mathbf{D}$ are optimization parameters. Optimization problem of each step is identical with that of light field stereoscopes[8], which could be solved by using simultaneous algebraic reconstruction technique (SART)[9]. The second step (i.e. update of $\mathbf{B}_k$), however, contains the binary constraint as described in the manuscript. For relaxation of the binary constraint, optimization target is multiplied by an integer constant T so that each pixel of backlight usually illuminate corresponding RGB pixel by T times. This modification enables us to adjust the gradation of backlight according to the number of illumination $(0, 1, ..., T)$. Using this modification, we could solve more intuitive least squares problem as follows.

$$\min \sum_{k=1}^{p^2} \left\| T\mathbf{V}_k - \sum_{j=1}^{m} \mathbf{P}_{(k,j)} \left(\mathbf{B}_j \odot \mathbf{D}\right) \right\| \approx T \min \sum_{k=1}^{p^2} \left\| \mathbf{V}_k - \sum_{j=1}^{m/T} \mathbf{P}_{(k,j)} \left(\overline{\mathbf{B}}_j \odot \mathbf{B}\right) \right\|, \tag{3}$$

where $T\overline{\mathbf{B}}_j$ should be integer. Note that larger T enhances the performance to represent occlusion boundary. However, it may degrade overall contrast since illumination time of each RGB pixel gets longer.

In this study, we solved the optimization problem on the left side. In order to solve this problem using SART, we ignore the binary constraint and update $\mathbf{B}_k$ for some iteration. Then, we calculate the backlight energy distribution of display pixels by adding up $\mathbf{B}_k$. The backlight energy distribution indicates that illumination time of each display pixels. Using the distribution map as a milestone, we convert $\mathbf{B}_k$ to an appropriate set of integers so that the changes of backlight energy distribution as well as errors between $\mathbf{B}_k$ and updated $\mathbf{B}_k$ are minimized. We call this process as regularization of backlight sequences, which is one of the most important step for convergence of this algorithm. After the regularization step, we go back to the first step to update $\mathbf{B}_k$ and repeat this procedure for some iterations. Finally, we may find approximated solution for the NP-hard problem.

In summary, we describe the algorithm with a pseudo-code in Algorithm 1. In this study, we set optimization constants as follows. $maxiter_1$ and $maxiter_2$ are 140 and 120, respectively. p, n, and m are 7, 450, and 80, respectively. c is 0.0, which is referred to as offset of FSAB. The integer constant, T, is assumed as 8.

Algorithm 1 Optimize backlight images $\mathbf{b}_1, \mathbf{b}_2, ..., \mathbf{b}_m$ and RGB image $\mathbf{D}$

Load $p \times p$ perspective views $\mathbf{V} = [\mathbf{v}_{r1}, \mathbf{v}_{r2}, ...\mathbf{v}_{rp^2}, ..., \mathbf{v}_{g1}, ..., \mathbf{v}_{bp^2}]^{\mathrm{T}}$ of size $3n^2p^2 \times 1$, $p \times p$ depth maps of size $n^2p^2 \times 1$, Projection matrix $\mathbf{P}$
Initialize Backlight images $\mathbf{B} = [\mathbf{b}_1, \mathbf{b}_2, ...\mathbf{b}_m]^{\mathrm{T}}$ of size $mn^2 \times 1$, Display RGB image $\mathbf{D} = [\mathbf{d}_r, \mathbf{d}_g, \mathbf{d}_b]^{\mathrm{T}}$ of size $3n^2 \times 1$.
for $iter = 1$ **to** $maxiter_1$ **do**
 $\mathbf{P}_1 = \mathbf{P} * \mathrm{diag}([\mathbf{B}^{\mathrm{T}}, \mathbf{B}^{\mathrm{T}}, \mathbf{B}^{\mathrm{T}}])$
 Update D: Solve $\min \|T\mathbf{V} - \mathbf{P}_1\mathbf{L}\|$ by using SART
 $\mathbf{D} > 1 = 1, \mathbf{D} < 0 = 0$
 if $iter < maxiter_2$ **then**
  $\mathbf{P}_2 = \mathbf{P} * ([\mathrm{repmat}(\mathbf{d}_r, [m, 1]); \mathrm{repmat}(\mathbf{d}_g, [m, 1]); \mathrm{repmat}(\mathbf{d}_b, [m, 1])])$
  Update B Solve $\min \|T\mathbf{V} - \mathbf{P}_2\mathbf{B}\|$ by using SART
  $\mathbf{B} > 1 = 1, \mathbf{B} < c = c$
  if $\mathrm{mod}(iter, 10) = 0$ **then**
   $\mathbf{B}_{new} = \mathrm{round}(\mathbf{B})$
   Regularize B $\mathbf{R} = \mathrm{sum}(\mathrm{reshape}(\mathbf{B} - \mathbf{B}_{new}, [n^2, m])), 2)$
   while $\mathbf{R} \neq 0$ **do**
    subtract or add 1 elements of $\mathbf{B}_{new}$ according to $\mathbf{R}$ and $\mathbf{B}$
   end while
  end if
 end if
end for

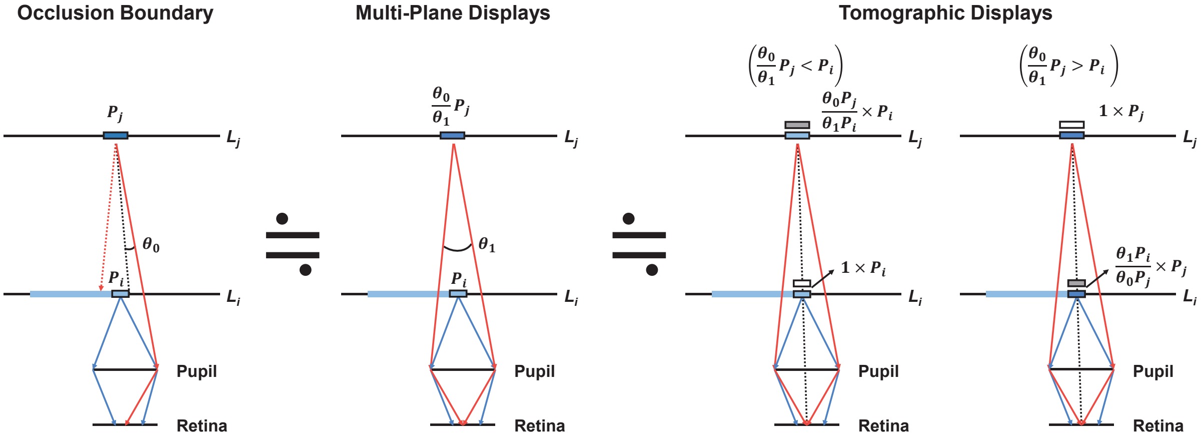

Supplementary Figure 3. Optical approximation for occlusion blending. Using this approximation, we could establish the initial condition of tomographic displays. Note that backlight images should be averaged for three colours unless backlight supports full colours via temporal multiplexing. Also, we ignored unnoticeable occlusion boundary between focal planes with small spacing.

Initial Condition Design

The primitive solver has a clear limitation that it works well only if we give appropriately determined initial condition. Without the initial condition, we usually observe that the iterative algorithm is trapped in a local minimum that shows poor performance. In order to avoid local minimum and enhance the optimization performance, we design the initial condition based on optical principle of multi-plane displays. Supplementary Figure 3 describes how multi-plane displays could mitigate occlusion boundary artifacts by using optimal blending[1]. As shown in the figure, there is an optical relationship between two plane images that represent occlusion boundary. Using this relationship, we could obtain the approximated solution for occlusion blending of tomographic displays.

Since the approximated solution of backlight images is not binary, conversion process is required. When P_i is larger than $\frac{\theta_0}{\theta_1}P_j$, the illumination time of corresponding pixel is determined by $T\frac{\theta_0 P_j}{\theta_1 P_i}$. Since each backlight pixel could not be larger than 1, the illumination time is divided into adjacent backlight images. Still, some pixels of backlight images could be larger than 1 because of the duplication. In the last step, we rearrange the backlight pixel values so that they satisfy binary constraint as well as boundary condition.

Additional Simulation and Experimental Results

Supplementary Figure 4 demonstrates convergence rates of our method, which shows validity of the optimization algorithm. If we solve the optimization problem as described in Algorithm 1, optimal RGB image and binary image sequences are obtained. Supplementary Figure 4 describes optimization results according to the blending method. As we can see in the experimental results, optimization blending enables tomographic displays to represent occlusion boundary with much less artifacts. According to the results, the proposed solver for NP-hard problem finds a convincing optimal solution that produces retinal images of high fidelity, which is comparable to that of multi-plane displays using optimal blending.

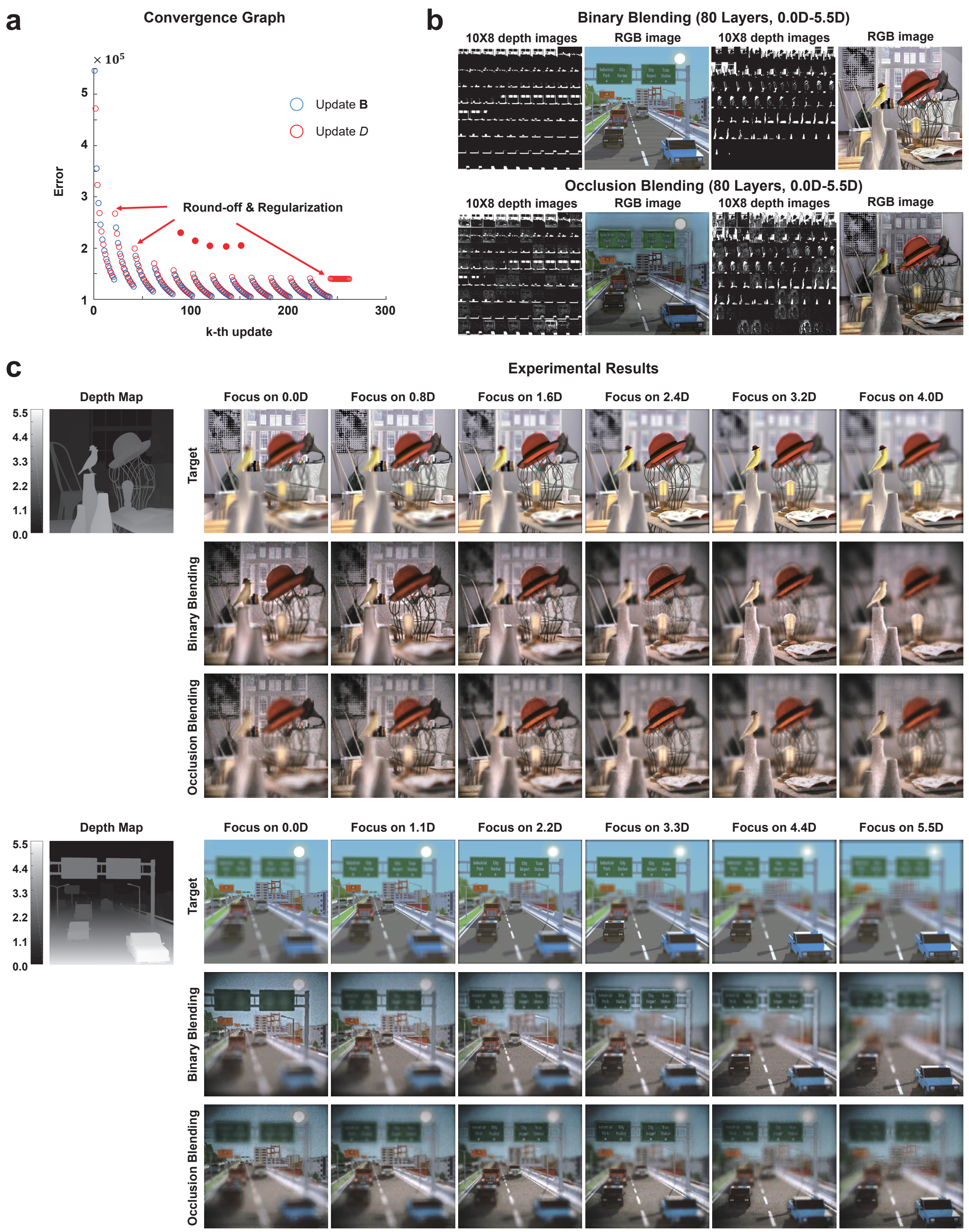

Supplementary Figure 4. Optimization results using occlusion blending. (a) Convergence graph of proposed algorithm is demonstrated. The graph shows our algorithm is converged on an optimal point. Note that the first blue circle indicates the error of the initial condition. (b) Focal plane images are presented according to the blending methods. Note that we visualize binary image sequences for FSAB by using a merged single image. (c) Experimental results are demonstrated according to the blending methods of tomographic displays. Occlusion blending shows enhanced performance to represent occlusion boundary.

Supplementary Note 3: Analysis of Depth Discontinuities

In this section, we will demonstrate how depth discontinuities are observed in tomographic displays. We also compare the degree of artificial effects with other related display systems, which verify the outstanding performance of tomographic displays. We could investigate the effect of depth discontinuities in terms of fidelity, contrast, and accommodation cues.

Depth Discontinuities Recognition of Human Vision

For analysis of depth discontinuities, we define line spread function of human visual system. Basically, human visual system consists of iris, eye-lens, and retina. Iris is a stop aperture of optical system, eye-lens is an optical lens to change focusing planes, and retina is a sensor to estimate brightness and colours. Thus, optical imaging of human visual system could be represented by waveoptics as follows[10].

$$H(f_X, f_Y) = P(\lambda z_d f_X, \lambda z_d f_Y) \exp[jkW(\lambda z_d f_X, \lambda z_d f_Y)], \tag{4}$$

where H denotes transfer function of human visual system, P is the Fourier transform of the pupil function, and W is phase distortion caused by optical imaging system. λ is wavelength of light source, z_d is distance between image point and exit pupil, and (f_X, f_Y) indicates Fourier domain.

The phase distortion W is usually considered as the focusing error, which is given by

$$W(x,y) = -\frac{1}{2}\left(\frac{1}{z_e} - \frac{1}{z_d}\right)\left(x^2 + y^2\right), \tag{5}$$

where z_e is focal depth of human visual system, and (x,y) indicates spatial domain. In practical sense, however, this equation is not exactly matched with the perception studies of resolution limit for human visual system. Human visual system has much less resolving power than theoretical estimation because of several reasons including spherical aberration and chromatic aberration. In order to consider these terms, we employ Seidel coefficients and achieve averaged line spread function over all visible wavelengths.

We determine the value of Seidel coefficient W_{040} via comparison between theoretical and experimental results. We assume that human visual system has resolution limit of 30cpd and perception limit of 2% variation[11]. According to the assumptions, the coefficient W_{040} is determined as -0.555λ. Using this coefficient and Supplementary Eq. 4, we could analyze how depth discontinuity is observed by human visual system.

Binary Blending to Alleviate Depth Discontinuities

Instead of applying linear blending to mitigate depth discontinuities, tomographic displays may employ an alternative approach (i.e. binary blending) that modifies binary image sequences and RGB images simultaneously. This method is a simplified linear blending where voxels between adjacent planes are reconstructed by three states: one of the two planes assigns full radiance of voxels, or both of the planes assigns half radiance of voxels. If half radiance of voxels is assigned, brightness of RGB images is modified in half while binary images are duplicated. In other words, this approach reconstructs additional focal plane between the adjacent planes to mitigate depth discontinuities.

Supplementary Figure 5 illustrates the binary blending method and comparison between related approaches. We simulated how observers perceive the synthesis of focal plane images based on the analysis of previous section. In this simulation, we assume that multi-plane display with dense focal planes is a reference system. Using the simulation results, we could analyze the artificial effect caused by depth discontinuities. We compare three candidates: multi-plane displays with 0.6D layer spacing, tomographic displays without depth processing, and tomographic displays with binary blending. Similar analysis could be found in a previous research[12].

According to the simulation results, we note that multi-plane displays mitigate artifacts caused by depth discontinuities. However, they could not provide accurate focus cues as well as high fidelity retinal images of high frequency (20 cpd). The contrast of 20 cpd information becomes lower when users focus on the mid-plane, which indicates multi-plane displays could not provide appropriate accommodation cues. On the other hand, tomographic displays with binary blending could provide higher fidelity retinal images of 20 cpd as well as more accurate accommodation cues. This result shows that tomographic displays have potential to support high resolution above 20 cpd, which was reported as difficult via multi-plane displays of 0.6D spacing using optimal blending[1].

We also simulate how pupil movement affects the synthesis of focal plane images. Supplementary Figure 6 demonstrates that pupil movement may degrade the image fidelity of tomographic displays. We can observe the pupil movement distortion that was not noticed in the experiment (Supplementary Fig. 2) due to the resolution limit. In addition, we recognize that binary blending enables tomographic displays to have some tolerance for the pupil movement. For more intuitive comparison and evaluation of tomographic displays with binary blending, simulation results using a 3D scene are presented in Supplementary

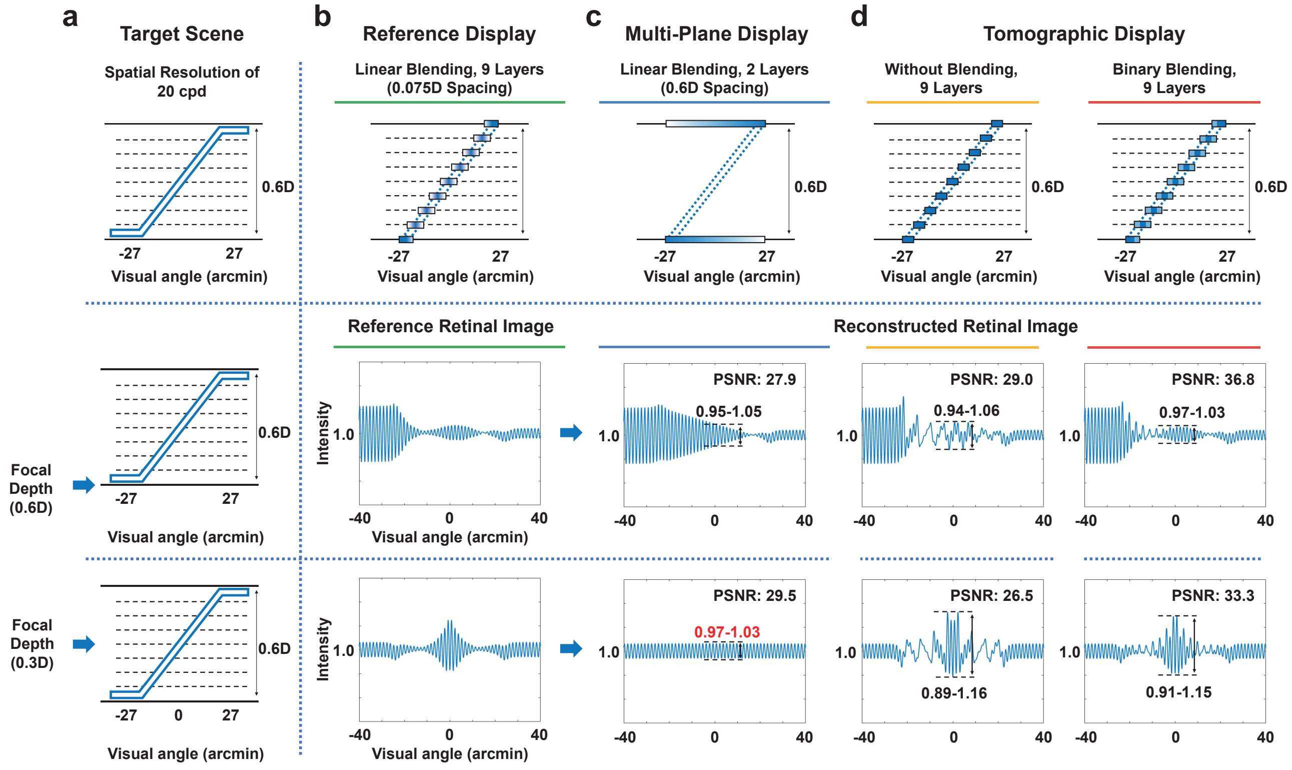

Supplementary Figure 5. Analysis of artifacts caused by depth discontinuities. (a) We suppose a slanted bar that covers depth range of 0.6D with a sinusoidal texture of 20 cpd. (b) We consider multi-plane display with dense focal planes (0.075D spacing) as a reference system. When the slanted bar is reconstructed by multi-plane or tomographic displays, the sinusoidal texture of 20 cpd is observed as described in composite retinal images. (c-d) As demonstrated in PSNR and retinal images, tomographic displays with binary blending could provide more accurate focus cues as well as high fidelity images. These results verify the validity of binary blending as well as merits of tomographic displays compared to multi-plane displays.

Fig. 7. As shown in the results, tomographic displays could provide higher resolution images with high frequencies. Binary blending alleviates the artifacts caused by misalignment of adjacent focal planes. Although binary blending could not resolve the occlusion boundary issues, it would be a convincing and efficient solution for real-time operation.

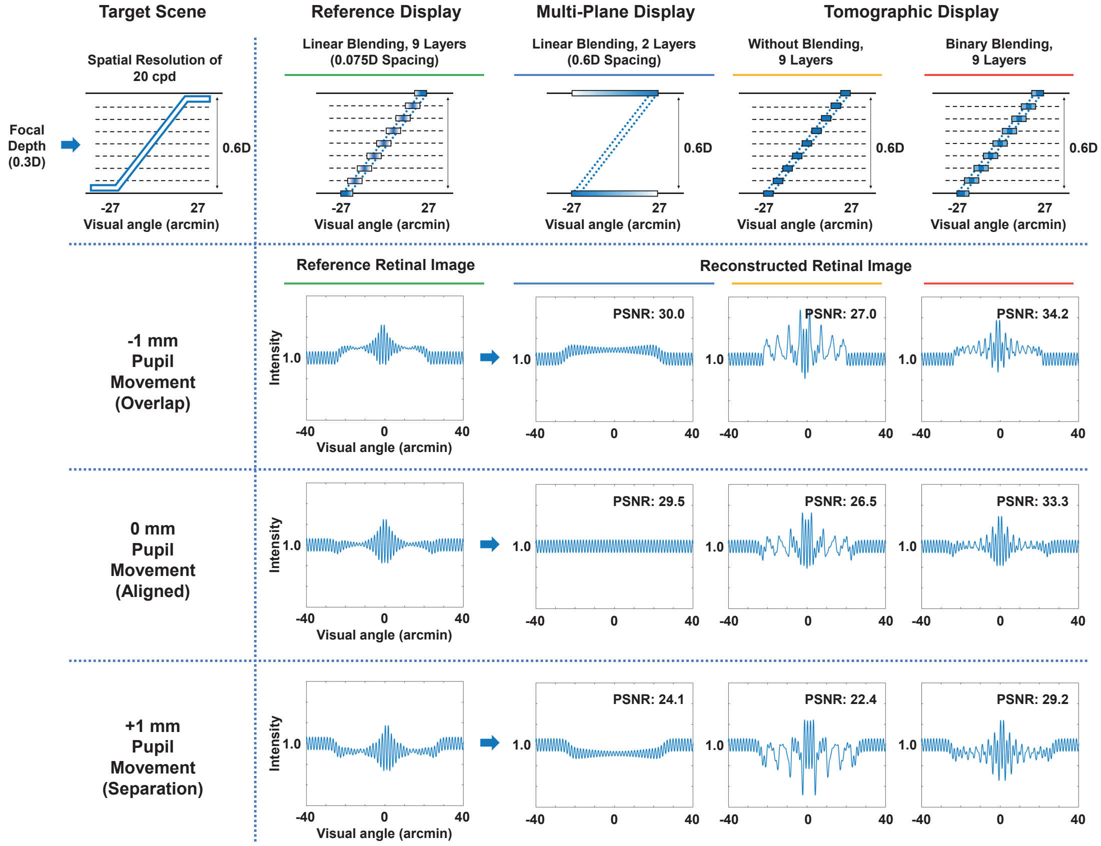

Supplementary Figure 6. Demonstration of pupil movement and depth discontinuities. We suppose a slanted bar that covers depth range of 0.6D with a sinusoidal texture of 20 cpd. When the slanted bar is reconstructed by multi-plane or tomographic displays, the sinusoidal texture of 20 cpd is observed as described in composite retinal images. Each row illustrates how retinal images are affected by the pupil movement (-1mm, 0mm, and 1mm). Note that we consider multi-plane display with dense focal planes (0.075D spacing) as a reference system. Tomographic displays with binary blending show the most convincing performance to provide finite retinal images of high fidelity.

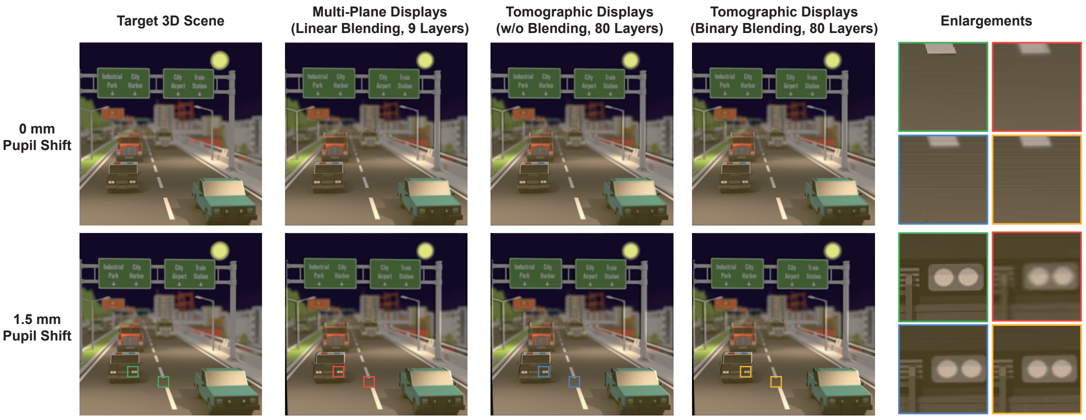

Supplementary Figure 7. Comparison and evaluation of binary blending using a 3D scene. The 3D scene has the depth range of 5.5D (18cm-infinity), which is observed by a 4mm pupil that focuses on the depth of 3.7D. On the right hand side, enlargements of images demonstrate the advantages of binary blending as well as tomographic displays. First, multi-plane displays could not provide high frequency information of 3D scenes. Second, binary blending alleviates the artifacts that occur when pupil is shifted by 1.5mm from the desired position.

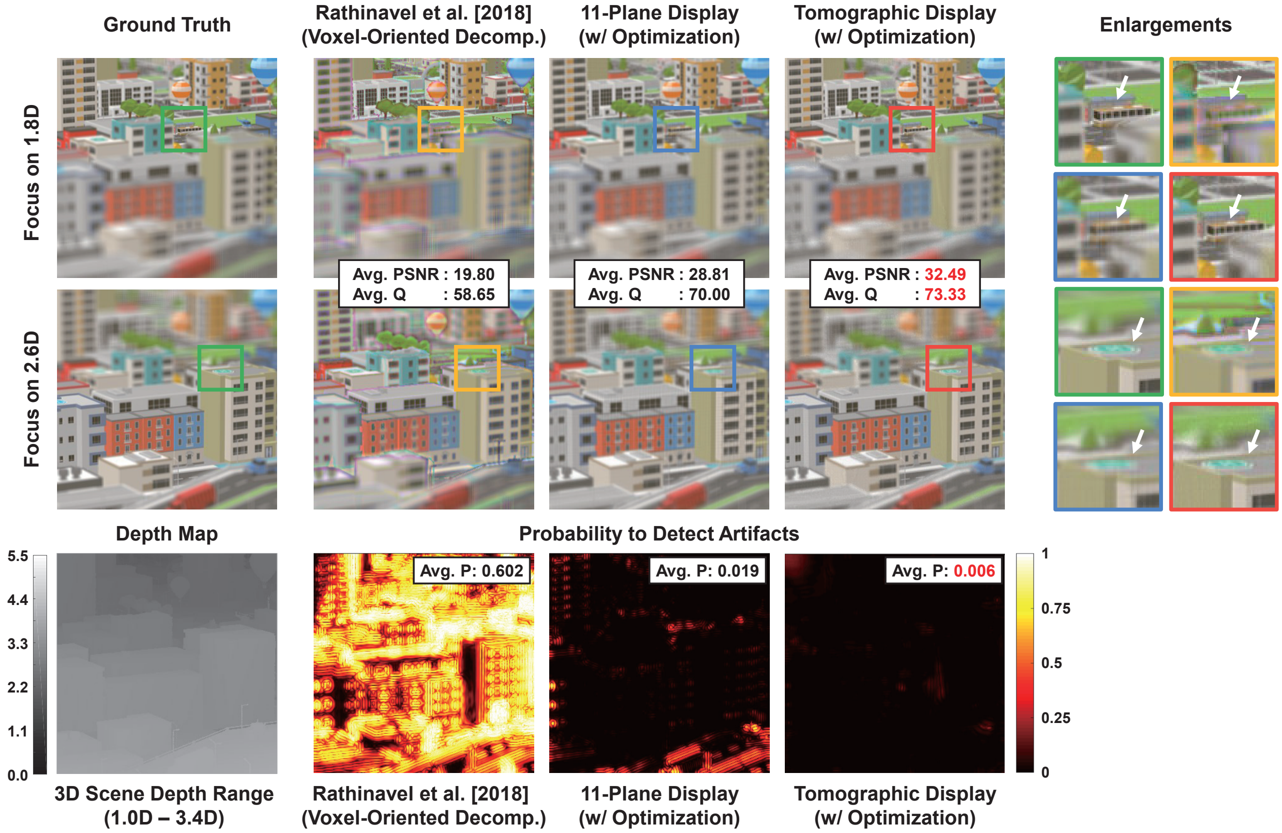

Supplementary Figure 8. Quantitative evaluation of tomographic displays. As shown in the results, tomographic displays enable users to observe the most accurate retinal images. Note that the performance of Rathinavel et al.'s system[2] is degraded by the chromatic distortion and artifacts at occlusion boundaries. If an adequate optimization algorithm is introduced in the future work, the performance is expected to be enhanced.

Supplementary Note 4: Additional Results for Evaluation of Tomographic Displays

In this section, we will demonstrate the competitive performance tomographic displays compared to following display systems: multi-plane displays of 11 layers with 0.6D spacing and voxel-oriented multi-plane displays of 280 layers[2]. Note that multi-plane displays of 11 layers are representative system for conventional multi-plane displays[1,12] where four planes are separated by 0.6D. For comparison, we apply the identical quantitative analysis introduced in the manuscript where all systems have resolution limit of 20 cpd where the horizontal field of view is set to 10°. Retinal images are derived by synthesizing 7 by 7 sampled multi-view images on the pupil plane. The pupil size of human eyes is set to 6mm. As demonstrated in Supplementary Figure 8, tomographic displays enable users to observe the most accurate scenes with minimized errors. Tomographic displays show the highest values of average peak signal noise ratio (Avg. PSNR), average quality factor (Avg. Q), and average probability to detect artifacts (Avg. P). Supplementary Figure 9 illustrates the focal plane images that are used for the comparison.

Supplementary Note 5: Specification of Prototypes

In this section, we will experimentally verify the specifications of presented prototype. We estimate the number of focal planes, offset luminance of the backlight diffuser, and field of view.

Field of View and Offset Luminance

As demonstrated in Supplementary Fig. 10, we could estimate field of view and offset luminance (c) caused by the backlight diffuser. First, our prototype supports 30° diagnoal field of view that is measured when all of backlight images and RGB image are white board. Second, offset luminance is estimated by illuminating a specific region of the LC panel while LC panel displays a white board. When the illumination time of the specific region is set to $T/80$, the illuminating region shows the brightness of $A_1 = I(c+T/80)$. Luminance of the other region is supposed to be $A_2 = Ic$. In this experiment, we measure luminance values, A_1 and A_2, to derive the degree of the offset luminance given by $c = (A_2T)/80(A_1 - A_2)$. As shown in Supplementary Fig. 10, c is measured as 0.025 where $A_1 = 0.095$ and $A_2 = 0.38$. Note that we take the average of three different conditions: $T = 3$, $T = 5$, and $T = 10$.

Reconstruction of 80 Focal Planes

As demonstrated in the manuscript, the implemented prototype supports 80 focal planes between 18cm (5.5D) and infinity (0.0D). In other words, each focal plane is separated by 0.07D. The 0.07D separation is too narrow to be observed by a c-mounted camera lens because the depth of field of the lens is larger than 0.07D. Thus, we employ a DSLR camera (Canon EOS 5D) with a 50mm lens (Canon 50mm 1:1.4 EF), which has higher numerical aperture. Although this camera could not capture full field of view of prototype, it is appropriate to estimate the number of layers.

Supplementary Figure 11 demonstrates the experimental results. In the experiment, the prototype reconstructs 80 points that are floated at different depths between 0.0D and 5.5D. For estimation of each point depth, we employ point spread function. Point spread function is estimated according to the depth of image plane as shown in the first row of Supplementary Fig. 11. Note that the focal length of focus-tunable lens is set as constant when we derive corresponding point spread function. Using this point spread function, we predict the depth of 80 reconstructed points. As shown in the results, the prototype reconstructs focal planes at desired depths with the convincing accuracy.

Although tomographic near-eye displays show convincing reliability of focal plane reconstruction, we may observe some error of focal plane depths. The error seems to be caused by slight mismatch in synchronization of focus tunable lens and FSAB due to the different latency of two devices. Nevertheless, NI board resolves the mismatch periodically so that the overall system does not accumulate the errors. Note that users rarely observe the error since human visual system cannot observe the high frequency vibration. In addition, we believe that this issue could be settled by introduction of feedback circuit in commercialization step.

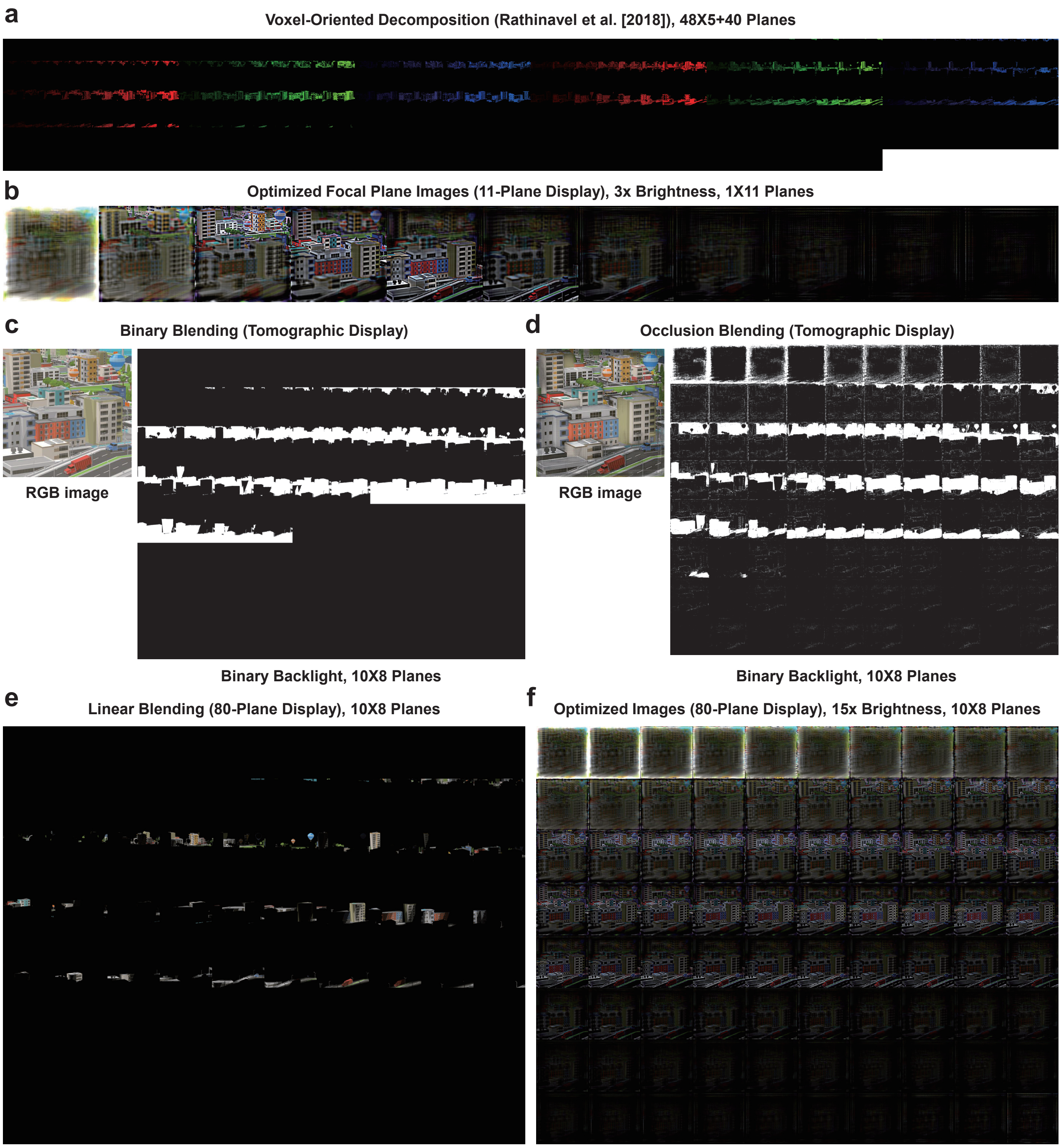

Supplementary Figure 9. Focal plane images used for retinal image simulation. (a) Voxel-oriented decomposed images and arbitrarily optimized images for Rathinavel et al.'s system[2]. (b) Optimized focal plane images for 11-plane displays[1,8]. (c) Binary and (d) Occlusion blended binary backlight seqeunces and RGB image for tomographic displays. (e) Linear blended and (f) optimized focal plane images for 80-plane displays.

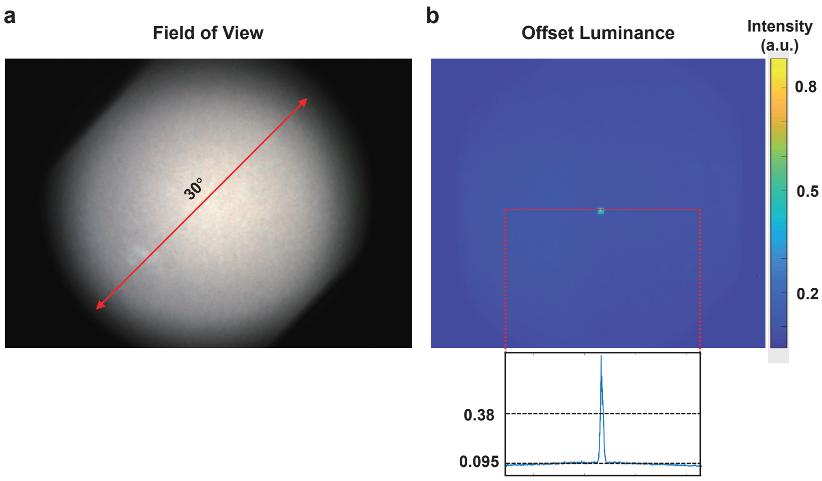

Supplementary Figure 10. Experimental results to estimate (a) field of view and (b) offset luminance.

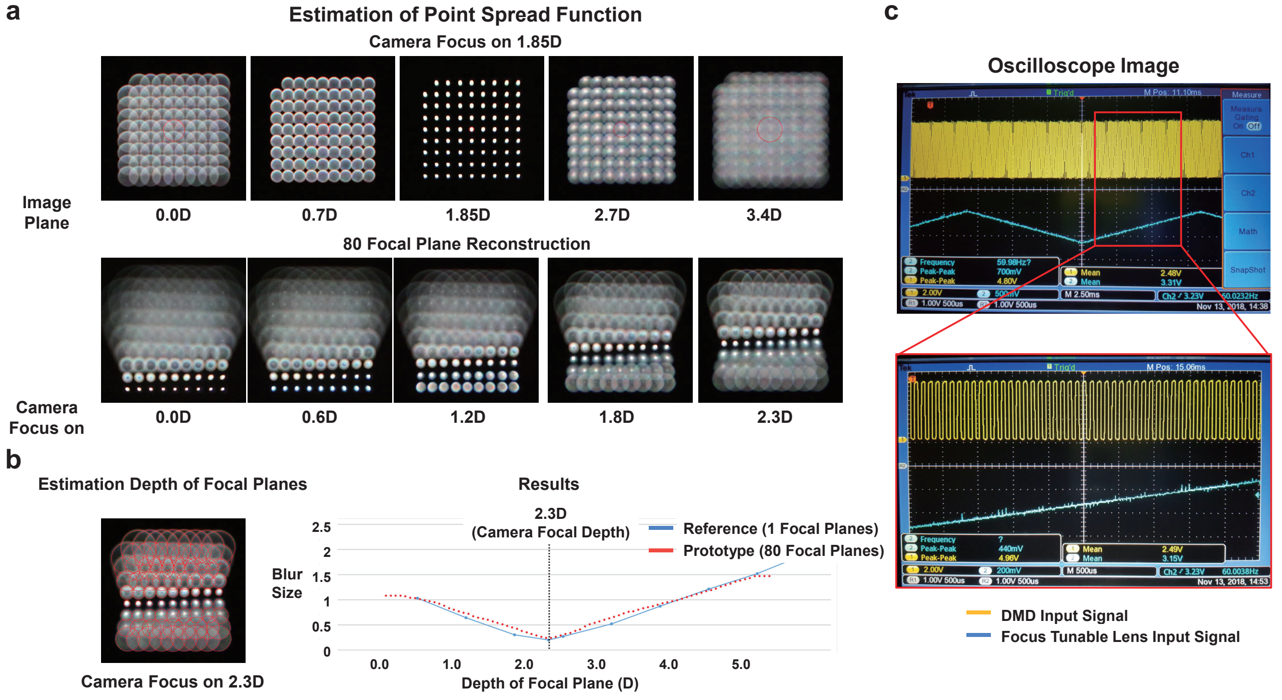

Supplementary Figure 11. Experimental results to estimate the depth of focal planes. (a) Point spread functions of the prototype according to the focal length of focus tunable lens are estimated. 80 reconstructed points are floated at different depths between 0.0D and 5.5D. (b) We estimate the blur size of each point, which is compared with point spread functions for prediction of reconstructed depths. Note that first 4 and last 4 points have the same depths because FSAB illuminates each point 8 times. (c) We captured oscilloscope images to show stability and synchronization of DMD and focus tunable signals.

Supplementary Note 6: Additional Results of Prototypes

Wearable Tomographic Near-Eye Displays Using a LED Array Backlight

As tomographic near-eye displays using DMD are too bulky for wearable near-eye displays due to the DMD projection system, we designed more compact system that employs a LED array backlight for FSAB. In order to show feasibility of wearable tomographic displays, we implemented a draft prototype using a LED array backlight. Supplementary Figure 12 describes two different systems of tomographic displays using either DMD or LED array. LED array backlight enables the form factor of tomographic displays to be compact enough for wearable displays.

LED array backlight, however, has limited refresh rate and spatial resolution compared to DMD backlight. The refresh rate of commercialized product provided by Adafruit is 500-800Hz. Thus, we could maximize the number of layers as 14 in laboratory environment. The small resolution could be a barrier to provide accurate accommodation cues. We believe that the specification of LED arrays could be improved in the near future as it would be easier than developing active LCD panels that operate at fast frame rate. In experiment, our prototype supports 8 backlight images with 8×8 resolution.

When FSAB is in lack of pixels or refresh rates, the depth map processing would enhance user experience. As demonstrated in Supplementary Fig. 13, low resolution depth map would have large depth discontinuities. For minimization of the artifacts at occlusion boundary, we could apply Gaussian filter to the original depth. If the number of focal plane is limited, layer spacing could be observed as occlusion boundaries. We note that the offset luminance of backlight could alleviate this seam effect. Thus, offset luminance is increased to $c = 0.2$ on purpose in LED array-based tomographic displays.

Supplementary Figure 14 demonstrates experiment results of the prototype using a LED array backlight. The prototype supports 8 focal planes with 0.8D layer spacing. Depth image has the resolution of 8×8 where the LED array has the pixel pitch of 3mm. We synchronize the tunable lens and the LED array backlight by using Arduino board. Display panel is identical model that is used for the DMD-based prototype. Experimental results show the feasibility of wearable prototype of tomographic displays.

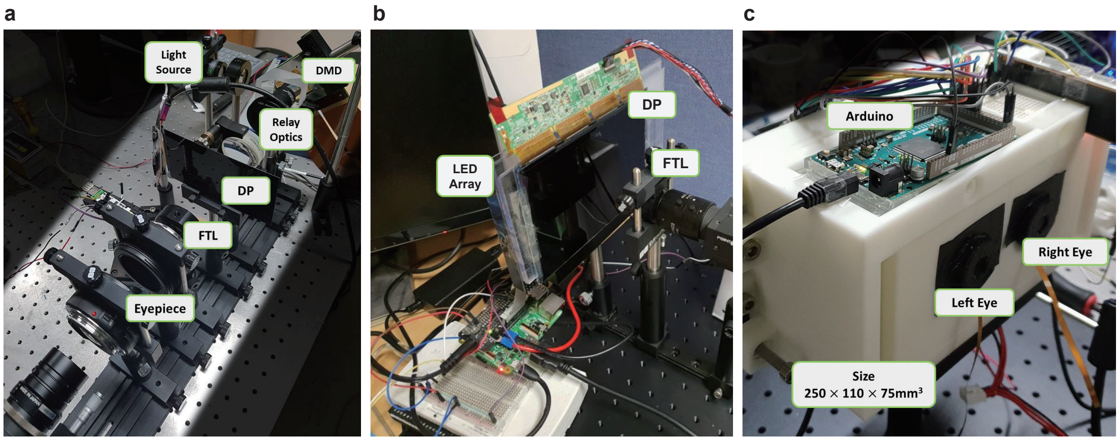

Supplementary Figure 12. Photographs of the prototypes. (a) We present a tomographic near-eye display that employs DMD projection system as FSAB. (b-c) We introduce a draft design of wearable prototype that uses a LED array (Adafruit 64×32 LED Matrix) as FSAB. The LED array could support 8×8 resolution and update a binary image at 480Hz, which can generate 8 tomographic layers.

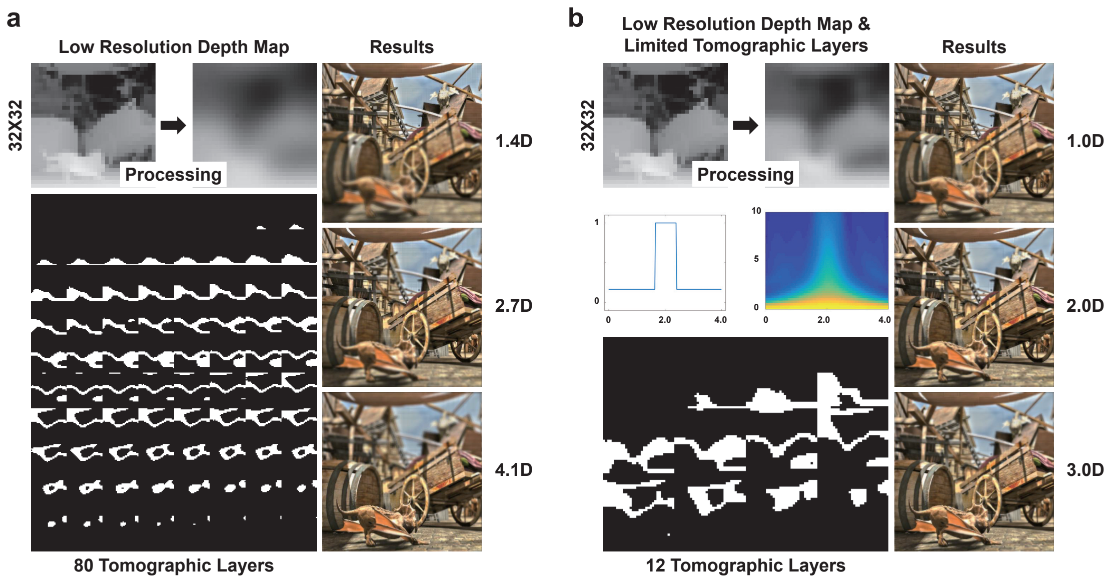

Supplementary Figure 13. Simulation results of tomographic displays using a LED array backlight. Compared to DMD projection, the LED array backlight has lower spatial resolution and secures less tomographic layers. (a) We describe simulation results when lower resolution backlight is used in the same environment with the prototype. (b) We simulate a condition when the backlight may only generate 12 tomographic layers. At this time, we apply more offset luminance ($c = 0.2$) and narrower depth of field to alleviate the seam effect. Note that the depth map is processed by using Gaussian filter in both cases. This image processing ensures the depth map is smoothly varying so that seam effect is minimized.

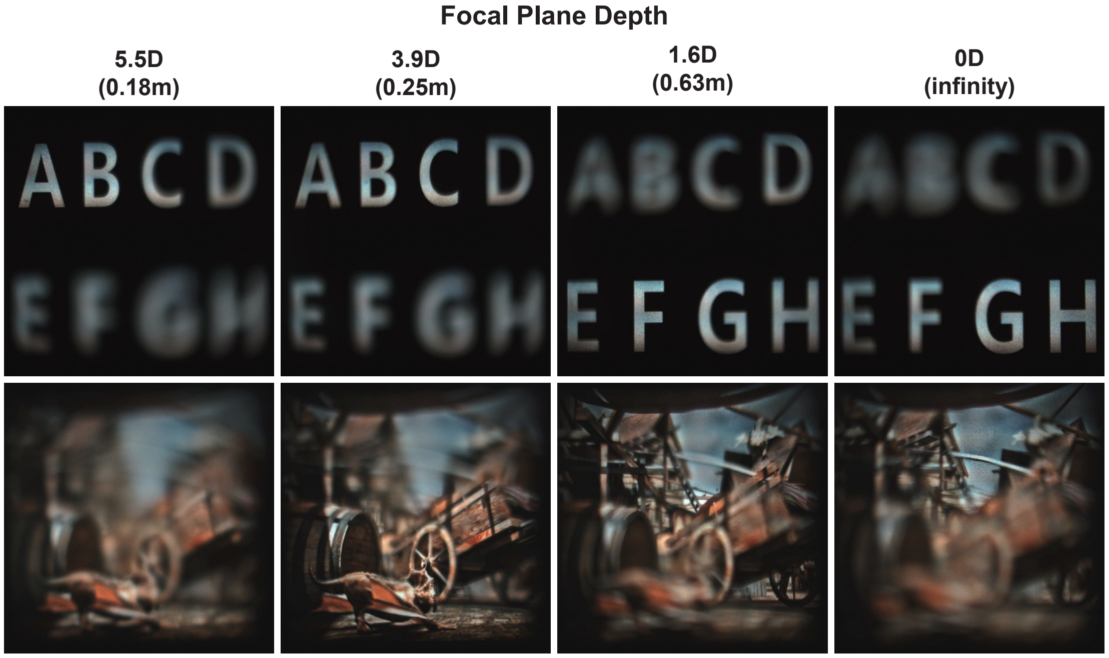

Supplementary Figure 14. Experimental results to show the feasibility of the wearable prototype that uses a LED array backlight. This system only supports 8 tomographic layers compared to the DMD-based prototype (80 layers) due to the limited refresh rate of the LED array backlight. Still, it shows superior performance among commercially available 3D display prototypes based on the conventional methodologies such as light field displays, multi-plane displays, or holographic displays.

Optical Specifications of the Benchtop Prototype

Supplementary Figure 15 illustrates the optical design for the benchtop prototype of tomographic near-eye displays. As illustrated in figure, our prototype has exit pupil of 7.5mm while providing field of view of 30°. According to the optical design, the prototype could cover depth of field between 0.0D and 10.5D. The horizontal size of the display panel is determined as 23mm. Note that camera lenses for eye-piece are approximated as Fresnel lenses with the same numerical aperture.

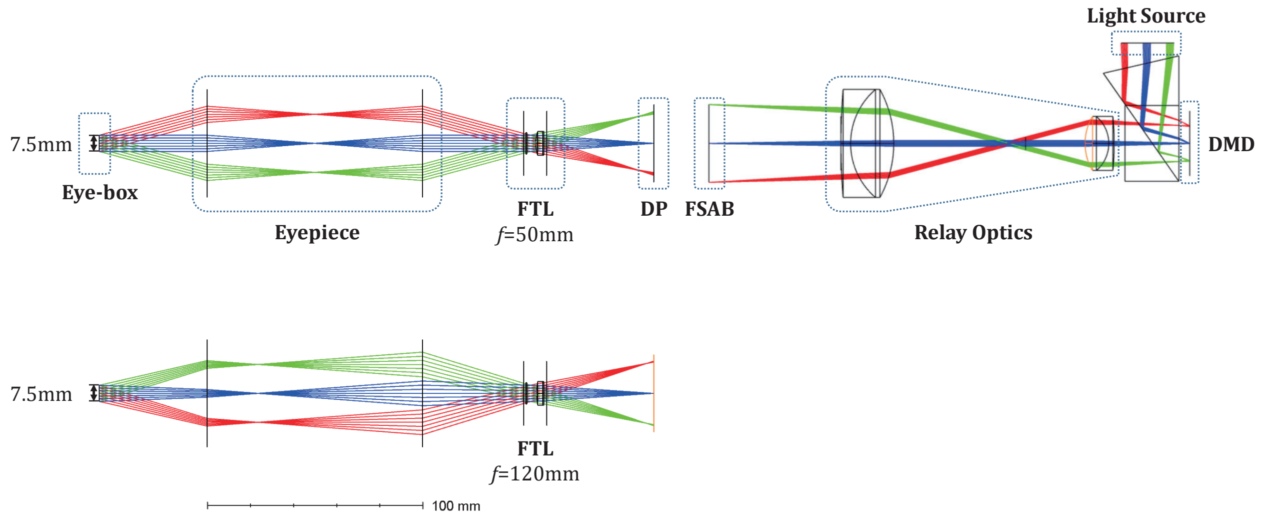

Supplementary Figure 15. Verification of optical design for the benchtop prototype using Zemax. According to Zemax simulation, exit pupil is estimated as 7.5mm; field of view is 30°×30°; the focal depth of display panel can be modulated between 10cm and infinity; and the display panel size is 23mm×23mm.

Supplementary Figure 16 demonstrates more experimental results of the implemented prototype to demonstrate motion parallax. We used two more 3D contents[13] that may show significant variation in the depth. Depth information of these contents are demonstrated in Supplementary Fig. 17 with the photographs to demonstrate continuous focus cues. We also compare the three illumination strategies: primitive, optimal, and HDR methods. The comparison results are shown in Supplementary Fig. 18. Because the primitive method has lower brightness than the optimal and HDR methods, we set higher gain for fair comparison. AS shown in the figure, optimal or HDR methods enable tomographic near-eye displays to provide higher contrast.

Supplementary Figure 19 demonstrates additional results of optimized illumination strategies. Supplementary Figure 20 illustrates backlight image sequences according to the illumination strategy when offset luminance is set to 0.05. The optimal illumination time is 10/480 seconds, which corresponds to 10 times illumination of display pixels during a single cycle. Supplementary Figure 20 also describes synchronization with focus-tunable lens and FSAB.

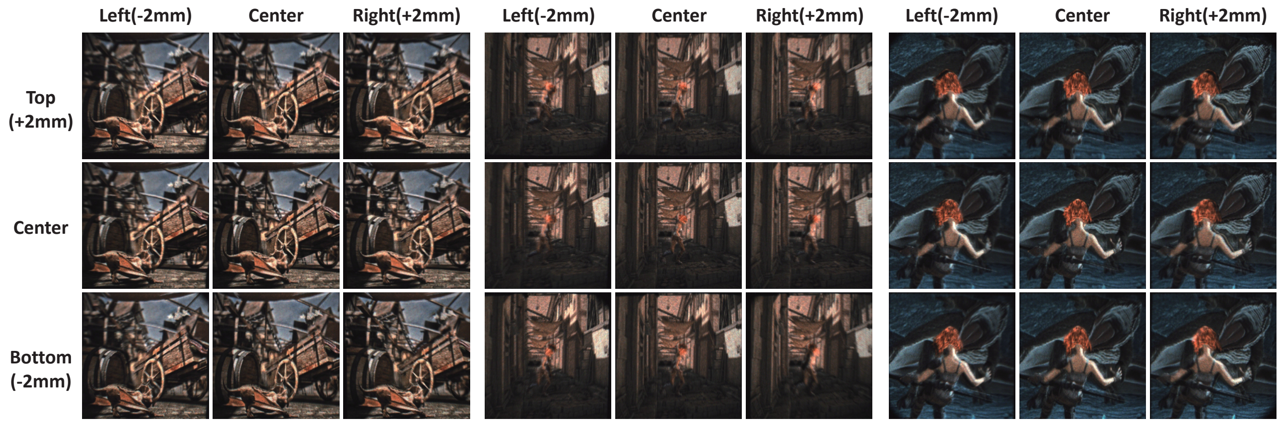

Supplementary Figure 16. Experimental results to demonstrate motion parallax.

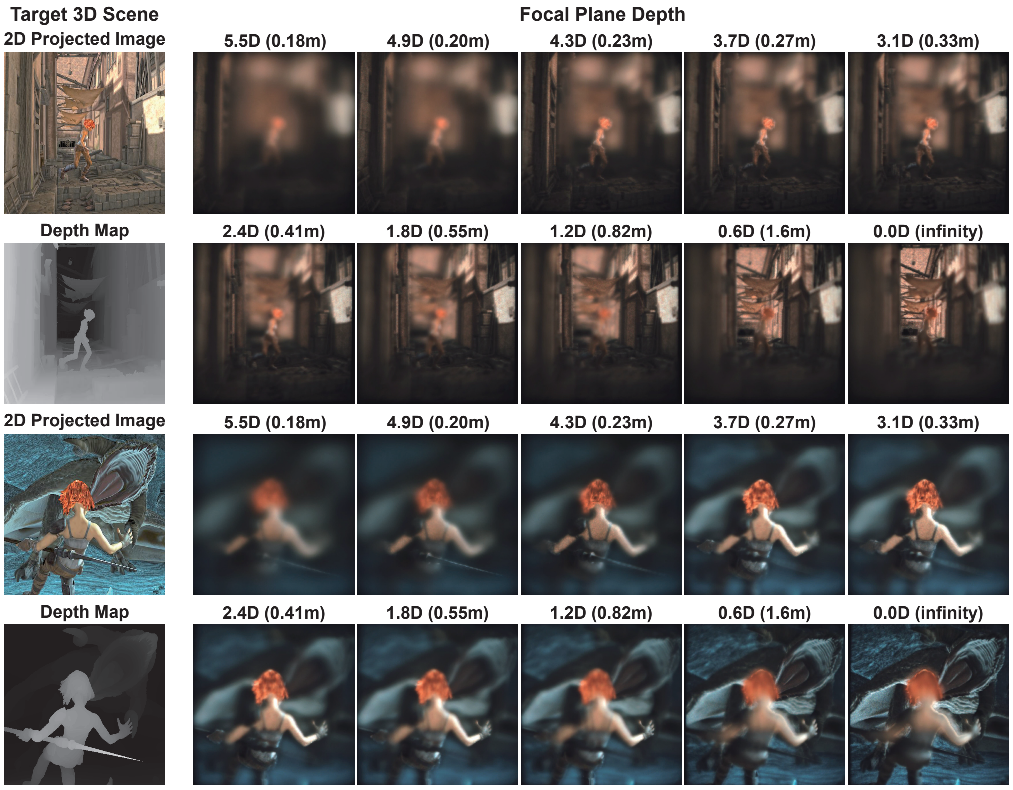

Supplementary Figure 17. Experimental results to demonstrate continuous focus cues.

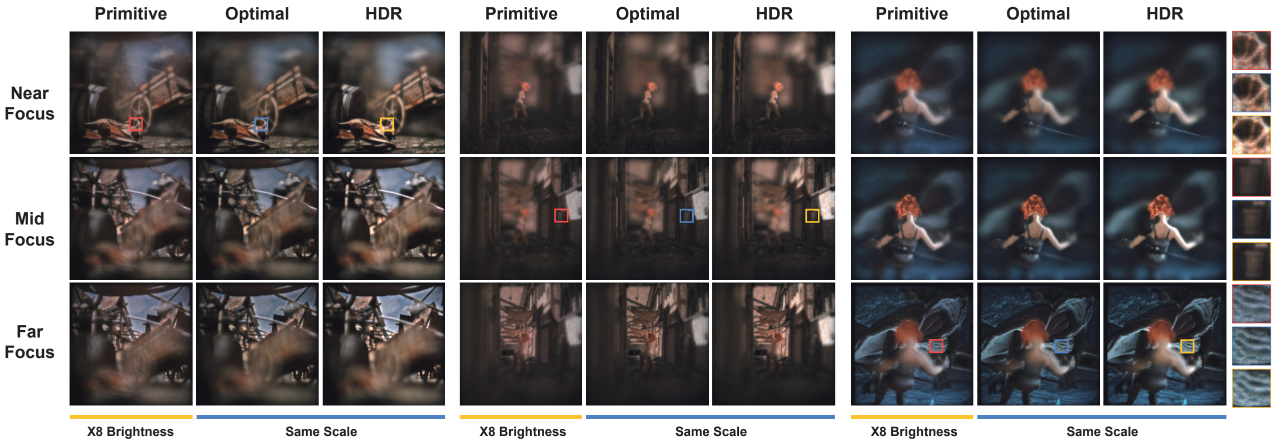

Supplementary Figure 18. Experimental results according to the illumination strategy. Note that the photographs for primitive method are captured in different specifications ($\times 8$ larger gain) of the CCD camera.

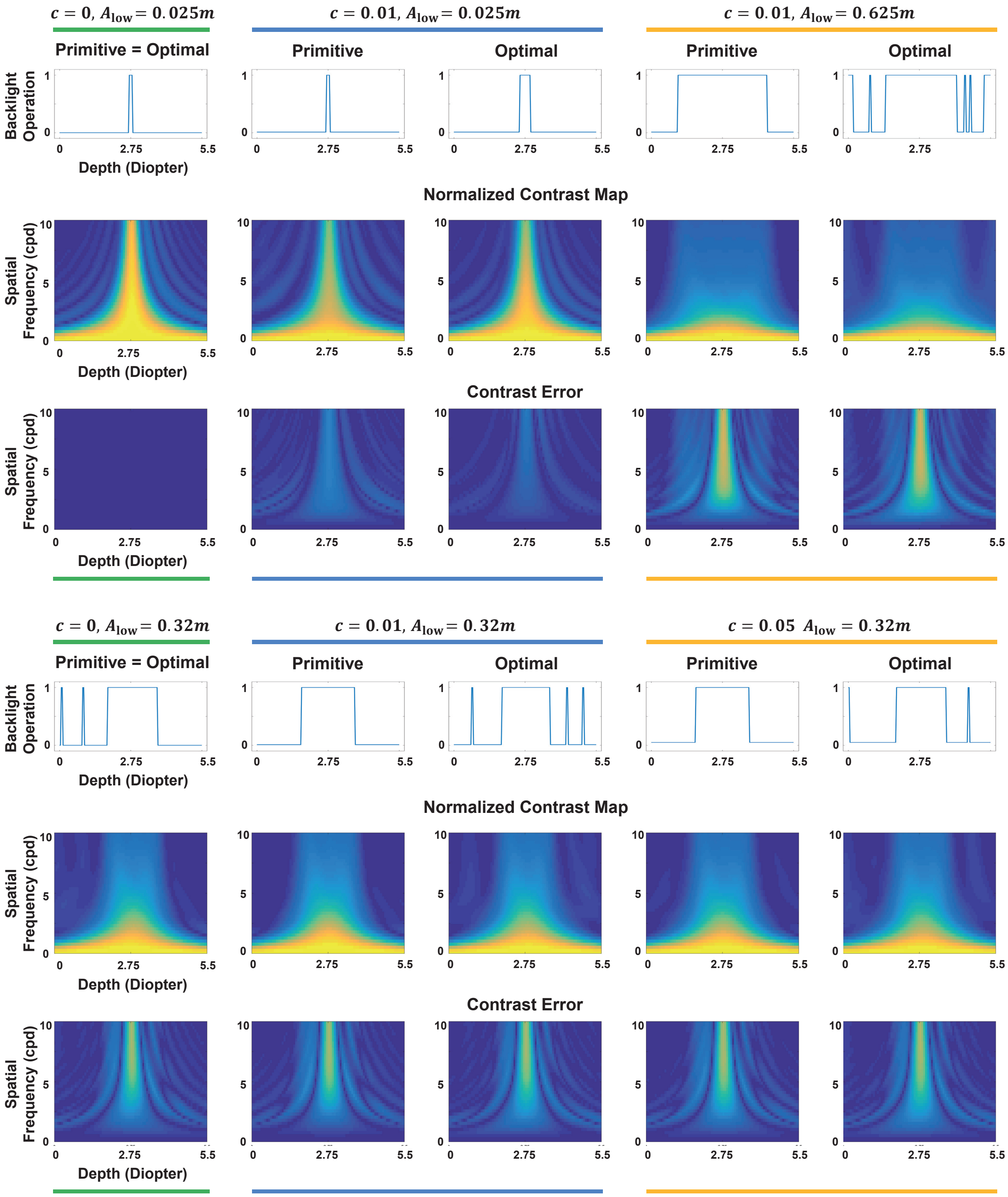

Supplementary Figure 19. Additional results of optimization for illumination strategy. We note that the second lobe of backlight operation also appears when low bound of brightness is $0.32m$.

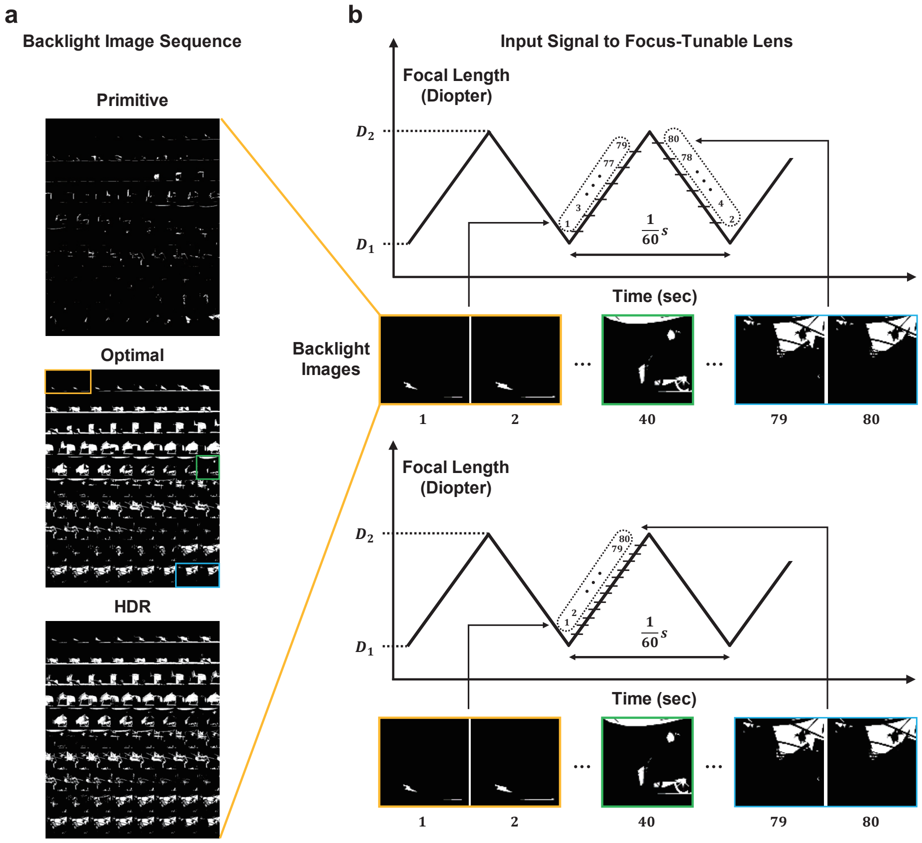

Supplementary Figure 20. Synchronization of the FSAB and the focus tunable lens. (a) We illustrate 80 tomographic layers according to the illumination strategies and 3D contents. (b) we present illustration to describe synchronization between a focus-tunable lens and a fast spatially adjustable backlight. The focus-tunable lens sweeps a specific dioptric range between D_1 and D_2 within a frame (1/60 second). During the single frame, 80 tomographic layers are sequentially reconstructed. As shown in the top row, adjacent layers could be crossly displayed to use full frame. However, this method involves stripe pattern noises as we discussed in previous section. We could insert black frame to ensure less noise.

Supplementary References

1. Narain, R. *et al.* Optimal presentation of imagery with focus cues on multi-plane displays. *ACM Trans. Graph.* **34**, 59 (2015).
2. Rathinavel, K., Wang, H., Blate, A. & Fuchs, H. An extended depth-at-field volumetric near-eye augmented reality display. *IEEE transactions on visualization computer graphics* **24**, 2857–2866 (2018).
3. Chang, J.-H. R., Kumar, B. & Sankaranarayanan, A. C. Towards multifocal displays with dense focal stacks. In *SIGGRAPH Asia 2018 Technical Papers*, 198 (ACM, 2018).
4. Rolland, J. P., Krueger, M. W. & Goon, A. A. Dynamic focusing in head-mounted displays. In *Stereoscopic Displays and Virtual Reality Systems VI*, vol. 3639, 463–471 (International Society for Optics and Photonics, 1999).
5. Lee, S. *et al.* Foveated retinal optimization for see-through near-eye multi-layer displays. *IEEE Access* **6**, 2170–2180 (2018).
6. Mercier, O. *et al.* Fast gaze-contingent optimal decompositions for multifocal displays. *ACM Trans. Graph.* **36**, 237 (2017).
7. Matsuda, N., Fix, A. & Lanman, D. Focal surface displays. *ACM Trans. Graph.* **36**, 86 (2017).
8. Huang, F.-C., Chen, K. & Wetzstein, G. The light field stereoscope: immersive computer graphics via factored near-eye light field displays with focus cues. *ACM Trans. Graph.* **34**, 60 (2015).
9. Andersen, A. H. & Kak, A. C. Simultaneous algebraic reconstruction technique (sart): a superior implementation of the art algorithm. *Ultrason. imaging* **6**, 81–94 (1984).
10. Goodman, J. W. Introduction to Fourier Optics. *4th ed., W.H. Free.* (2017).
11. Blackwell, H. R. Contrast thresholds of the human eye. *JOSA* **36**, 624–643 (1946).
12. Akeley, K., Watt, S. J., Girshick, A. R. & Banks, M. S. A stereo display prototype with multiple focal distances. *ACM Trans. Graph.* **23**, 804–813 (2004).
13. Butler, D. J., Wulff, J., Stanley, G. B. & Black, M. J. A naturalistic open source movie for optical flow evaluation. In A. Fitzgibbon et al. (Eds.) (ed.) *European Conf. on Computer Vision (ECCV)*, Part IV, LNCS 7577, 611–625 (Springer-Verlag, 2012).
