# Supplementary material for: Tomographic near-eye displays
Source: Nat Commun. 2019 Jun 7;10:2497. doi: 10.1038/s41467-019-10451-2 (PMC6555831; doi:10.1038/s41467-019-10451-2)
Supplement: Supplementary file 3 — Description of Additional Supplementary Files [file 41467_2019_10451_MOESM3_ESM.pdf]

Description of Additional Supplementary Files

File Name: Supplementary Movie 1

Description: Highlight video of tomographic near-eye displays. The video presents the intuitive description of tomographic near-eye displays, continuous focus cue reconstruction, capability of motion parallax, and full frame 3D scene movie.
